# The neuropsychological profiles and semantic-critical regions of right semantic dementia

**DOI:** 10.1016/j.nicl.2018.05.035

**Published:** 2018-05-29

**Authors:** Keliang Chen, Junhua Ding, Biying Lin, Lin Huang, Le Tang, Yanchao Bi, Zaizhu Han, Yingru Lv, Qihao Guo

**Affiliations:** a Department of Neurology, Huashan Hospital, Fudan University, Shanghai, China; b State Key Laboratory of Cognitive Neuroscience and Learning, IDG/McGovern Institute for Brain Research, Beijing Normal University, Beijing, China; c Department of Radiology, Huashan Hospital, Fudan University, Shanghai, China

**Keywords:** Semantic dementia, Lesion-behavior mapping, Laterality of brain atrophy, Semantic deficits

## Abstract

**Introduction:**

Previous literature has revealed that the anterior temporal lobe (ATL) is the semantic hub of left-sided or mixed semantic dementia (SD), whilst the semantic hub of right-sided SD has not been examined.

**Methods:**

Seventeen patients with right-sided SD, 18 patients with left-sided SD and 20 normal controls (NC) underwent neuropsychological assessments and magnetic resonance imaging scans. We investigated the relationship between the degree of cerebral atrophy in the whole brain and the severity of semantic deficits in left and right-sided SD samples, respectively.

**Results:**

We found the semantic deficits of right-sided SD patients were related to bilateral fusiform gyri and left temporal pole, whilst the left fusiform gyrus correlated with the semantic performance of left-sided SD patients. Moreover, all the findings couldn't be accounted for by total gray matter volume (GMV) or general cognitive degradation of patients.

**Discussion:**

These results provide novel evidence for the current semantic theory, that the important regions for semantic processing include both anterior and posterior temporal lobes.

## Highlights

• Left SD presented more impairment on the naming, reading and Stroop tests.
• Right SD exhibited more deficits on the symbol digit modalities task.
• Left fusiform gyrus correlated with the semantic performance of left SD.
• The semantic hubs of right SD were bilateral fusiform gyri and left temporal pole.

## 1 Introduction

Semantic dementia (SD) is a variant of progressive primary aphasia which is characterized by the specific semantic loss and preserved abilities of other cognitive functions (Gorno-Tempini et al., 2011; Hodges and Patterson, 2007). Its typical neuroanatomical feature is severe brain atrophy of the anterior temporal lobes (ATL) in both hemispheres (Gorno-Tempini et al., 2004a; Mummery et al., 2000).

According to the predominant atrophy hemisphere, this disorder can be split into two sub-types: left and right-sided SD. Their difference not only exists in the atrophy pattern, but also in the neuropsychological performance. Left-sided SD patients exhibit more naming and comprehension changes, whereas right-sided SD individuals suffer from more behavioral and face recognition problems (Brambati et al., 2009; Josephs et al., 2009; Seeley et al., 2005; Thompson et al., 2003).

Nevertheless, compared with left-sided SD, patients with right-sided SD are relatively rare. A study of a large series of consecutive SD patients found that only 25% cases were predominantly right-sided (Hodges et al., 2010). Therefore, sample size is a common limitation for research investigating right-sided SD. Until now, most research are still case studies (Brambati et al., 2009; Gorno-Tempini et al., 2004b; Joubert et al., 2004; Seeley et al., 2005; Snowden et al., 2012) and investigations with small samples (Kamminga et al., 2015; Kumfor et al., 2016). Only a few studies recruited big samples of right-sided SD (Binney et al., 2016; Chan et al., 2009; Hodges et al., 2010; Snowden et al., 2017). For example, Chan et al. (2009) collected 20 right-sided SD patients and compared their imaging and neuropsychological data with left-sided SD patients. Although these studies are excellent, further work is needed to use comprehensive assessments and voxel-based brain analyses to gain a better understanding of right-sided SD patients. Moreover, severity-matched groups and mild cases would be also required.

In fact, SD is thought to be direct evidence for the hub-plus-spoke model, which emphasizes the vital role of the ATL in semantic processing (Patterson et al., 2007; Ralph et al., 2017). Considerable studies have explored the semantic hub in SD individuals (Ding et al., 2016; Mion et al., 2010). Using strict regressions, they have demonstrated that the fusiform gyrus underpinned SD's semantic deficits. It's important to note that these studies only used left-sided or mixed SD cohorts, which might miss the chance to identify other areas. For example, floor and ceiling effects would occur in the left temporal pole and right fusiform gyrus, respectively. Indeed, right-sided SD patients also suffer from severe semantic deficits. Thus, investigating these patients would resolve the above issue and contribute to the understanding of semantic theory. To our knowledge, the semantic hub of right-sided SD has not been systematically examined.

In our study, we applied comprehensive neuropsychological assessments and voxel-wised imaging scans in 17 cases of mild right-sided SD and 18 cases of mild left-sided SD with comparable severity. Then we explored the neuropsychological deterioration, atrophy pattern and semantic-related areas of these two groups. We assumed that (1) both groups would present with severe semantic deficits; (2) left-sided SD group would present with more severe language problems than right-sided SD group; (3) in right-sided SD sample, the semantic-related regions would include other regions such as the temporal pole beyond the fusiform gyrus. In summary, by using a big sample of right-sided SD patients, our study identifies their comprehensive characteristics and provides new evidence for the semantic model.

## 2 Method

### 2.1 Subjects

Thirty-five SD patients were identified from the memory disturbance clinic of neurology department at Huashan hospital, Shanghai. The inclusion criteria included: reaching the current diagnostic criteria of SD (Gorno-Tempini et al., 2011), mild severity (MMSE >18), >6 years of education and completing neuropsychological assessments and MRI scans. Moreover, we measured the severity of white-matter hyperintensity through the Fazekas Scale (Fazekas et al., 1987) using T2 images. All subjects' periventricular hyperintensity (PVH) scores and deep white matter hyperintensity (DWMH) scores were ≤1. Thus, no subjects were excluded for the white-matter hyperintensity.

Twenty normal controls (NC) were recruited from the community, whose age, gender and education were matched with patients. All subjects were right-handed (measured by Edinburgh Handedness Inventory; Oldfield, 1971), native Chinese speakers with normal or corrected audition and vision and no psychiatric disease. Informed consent was obtained from all individual participants.

### 2.2 Neuropsychological tests

All subjects underwent routine clinical assessments (Guo and Hong, 2013) including domains of general cognitive function (MMSE & Memory and Executive Screening), episodic memory (Auditory Verbal Learning Test & Rey-Osterrich Complex Figure Test: long-delayed recalling), language (Similarity test, Boston naming test & Animal Verbal Fluency Test), attention (Symbol Digit Modalities Test), working memory (Digital Span Test), executive function (Trail Making Test & Stroop Color-Word Test), visuospatial skills (Rey-Osterrich Complex Figure Test: copy & Point Size Judgment Test), social cognitive function (Reading the Mind in the Eyes Test) and calculation (Exact Calculation, Magnitude Comparison & Proximity Judgment; see Table 1).

**Table 1:** Demographic and neuropsychological profiles of left and right-sided SD patients.

	Right SD	Left SD	NC	F	P
Age (years)	62.71 ± 7.75	61.33 ± 7.46	60.50 ± 3.95	0.532	0.591
Gender (Male:female)	6:11	9:9	8:12	χ^2^ = 0.820	0.664
Education (years)	11.35 ± 2.71	12.11 ± 3.31	10.45 ± 2.89	1.478	0.237
Course (years)	2.47 ± 2.12	3.00 ± 1.75	–	*t* = 0.807	0.426
General cognition					
MMSE (max = 30)	22.88 ± 3.72	21.17 ± 4.42	28.10 ± 1.37^b^, ^c^	22.001	<0.001
MES (max = 100)	58.00 ± 12.55	53.11 ± 12.22	82.85 ± 12.98^b^, ^c^	30.647	<0.001

Episodic memory					
AVLT-delay recall (n = 12)	0.94 ± 1.48	0.56 ± 1.20	5.75 ± 2.31^b^, ^c^	51.697	<0.001
CFT-memory (max = 36)	9.06 ± 4.87	10.22 ± 8.15	16.55 ± 6.57^b^, ^c^	6.889	0.002

Language					
ST (max = 20)	5.82 ± 4.29	3.94 ± 3.88	14.75 ± 2.73^b^, ^c^	47.879	<0.001
BNT (*n* = 30)	7.94 ± 4.07^a^	5.06 ± 3.52	22.10 ± 3.28^b^, ^c^	121.918	<0.001
AVFT (number in 60 s)	6.94 ± 3.70	5.72 ± 3.86	16.00 ± 3.66^b^, ^c^	43.192	<0.001

Attention					
SDMT (number in 90 s)	28.71 ± 11.47	34.44 ± 10.29	38.90 ± 10.80^b^	4.060	0.023

Working memory					
DST-forward (*n* = 12)	7.57 ± 1.34	7.08 ± 1.68	8.42 ± 1.38^c^	5.357	0.008
DST-backward (*n* = 10)	4.64 ± 1.28	3.42 ± 1.88	4.17 ± 1.27^b^	4.551	0.015
DST-order (n = 12)	4.29 ± 1.54	3.83 ± 1.85	5.00 ± 0.95^c^	4.721	0.013

Executive function					
SCWT-C_time_ (seconds)	104.00 ± 37.44	127.22 ± 37.52	78.50 ± 34.84^b^, ^c^	8.447	0.001
SCWT-C_accuracy_ (*n* = 50)	42.29 ± 6.91^a^	37.06 ± 11.39	48.10 ± 1.92^b^, ^c^	9.916	<0.001
TMT part B-A (seconds)	120.76 ± 58.78	114.00 ± 59.85	91.35 ± 36.05	1.643	0.201

Visuospatial perception					
CFT-copy (max = 36)	32.41 ± 2.74	32.78 ± 4.66	34.25 ± 2.02	1.643	0.203
PSJT (n = 30)	27.29 ± 2.05	27.94 ± 1.51	27.25 ± 1.62	0.919	0.405

Social cognition					
RMET (n = 30)	14.64 ± 4.72	15.47 ± 5.33	22.85 ± 2.94^b^, ^c^	22.094	<0.001

Arithmetic					
EC (n = 7)	6.24 ± 0.83	6.61 ± 0.85	6.50 ± 0.69	1.045	0.359
MC (n = 3)	2.88 ± 0.33	3.00 ± 0.00	3.00 ± 0.00	2.395	0.101
PJ (n = 3)	2.65 ± 0.61	2.61 ± 0.70	2.80 ± 0.41	0.577	0.565

Confrontation naming					
Picture naming (*n* = 140)	58.18 ± 25.34^a^	37.22 ± 28.40	125.65 ± 7.20^b^, ^c^	85.162	<0.001
Sound naming (*n* = 36)	8.53 ± 5.37	9.22 ± 5.61	26.95 ± 4.48^b^, ^c^	78.424	<0.001

Single**-**word comprehension					
Picture matching (*n* = 70)	51.88 ± 5.05	51.33 ± 8.97	66.45 ± 2.39^b^, ^c^	38.755	<0.001
Word matching (n = 70)	55.24 ± 6.42	50.83 ± 9.84	67.15 ± 1.46^b^, ^c^	30.232	<0.001
Word-picture verification (n = 70)	46.41 ± 12.57	41.00 ± 18.88	67.25 ± 1.94^b^, ^c^	22.085	<0.001

Object knowledge for low-frequency concepts					
Naming to definition (n = 70)	23.31 ± 11.33^a^	13.67 ± 13.25	58.40 ± 5.77^b^, ^c^	99.496	<0.001

Face knowledge					
Facial verification (*n* = 36)	19.00 ± 6.50	22.89 ± 7.39	28.15 ± 4.99^b^, ^c^	9.803	<0.001

Repetition					
Oral repetition (n = 12)	11.53 ± 0.51	11.22 ± 1.06	11.80 ± 0.41	3.098	0.054

Surface dyslexia					
Word reading (n = 140)	121.00 ± 21.63^a^	104.11 ± 31.72	137.85 ± 2.08^b^, ^c^	11.375	<0.001
Regularization errors (max = 12)	1.59 ± 1.37	2.06 ± 1.39	0.40 ± 0.75^b^, ^c^	9.815	<0.001

Grammar processing					
Picture description (accuracy)	0.91 ± 0.12	0.90 ± 0.12	0.91 ± 0.13	0.042	0.958

Note: Data are expressed as mean ± standard deviation.

MMSE = mini-mental state examination, MES = memory and executive screening, AVLT = auditory verbal learning test, CFT = Rey-Osterrich complex figure test, ST = similarity test, BNT = Boston naming test, AVFT = animal verbal fluency test, SDMT = symbol digit modalities test, DST = digital span test, NC = normal control, TMT = trail making test, SCWT = Stroop color-word test, PSJT = point size judgment test, RMET = reading the mind in the eyes test, EC = exact calculation, MC = magnitude comparison, PJ = proximity judgment, SD = semantic dementia.

a Right SD versus left SD: *p* < 0.05.

b Right SD versus NC: p < 0.05.

c Left SD versus NC: *p* < 0.05.

In addition, a comprehensive battery was used to examine semantic and non-semantic functions (Chen et al., 2017; Ding et al., 2016), including picture naming, facial verification, sound naming, naming to definition, picture associative matching, word associative matching, word-picture verification, word reading, repetition and picture description (see Table 1 and Supplementary material for details).

### 2.3 Image acquisition

Subjects were scanned in a 3 T MAGNETOM Verio MRI scanner. MPRAGE T1 weighted images were obtained with the following parameters: sagittal orientation, repetition time = 2300 ms, echo time = 2.98 ms, flip angle = 9°, matrix size = 240 × 240,field of view = 240 × 256 mm, slice number = 192, slice thickness = 1 mm, voxel size = 1 × 1 × 1 mm^3^.

### 2.4 Image preprocessing

T1-weighted images were first resampled to 1.5 × 1.5 × 1.5 mm^3^ and segmented into gray matter, white matter and cerebrospinal fluid using SPM8 (http://www.fil.ion.ucl.ac.uk/spm/). Next, images were normalized into the Montreal neurological institute (MNI) space. Then, gray matter volume (GMV) images were generated via affine transformation and non-linear warping, and smoothed using an 8-mm full-width at half-maximum Gaussian kernel. One patient was excluded due to the poor image quality.

### 2.5 Classification of patients

We first divided SD patients into left and right-sided groups according to the atrophy degree of bilateral ATLs qualitatively and then verified our classification results with the laterality index, which was evaluated by the formula: (Right ATL GMV- Left ATL GMV)/(Right ATL GMV + Left ATL GMV). The ATL was defined by the regions of temporal poles in the Automated Anatomical Labeling (AAL) Atlas (Tzourio-Mazoyer et al., 2002). A positive index value indicates a patient suffers from left-sided SD, and vice versa. Specifically, one patient was classified as left-sided SD by visual inspection due to bad quality of her T1-weighted image.

### 2.6 Statistical analyses of demographic and neuropsychological variables

We used SPSS19.0 (IBM Corp., Armonk, NY) to carry out these analyses. One-way analyses of variance were employed to reveal the differences among left, right-sided SD and NC groups. Then, we adopted post-hoc comparisons with the least significant difference (LSD) method. Specifically, gender was compared using a Chi-square test.

To measure the semantic performance of patients, principle component analysis (PCA) was conducted across all the battery tasks in left and right-sided SD groups respectively. We only extracted the factors whose eigenvalues were >1, rotated them using the varimax method and calculated factor scores with the regression model. The factor score with high loadings on semantic tasks was considered as the semantic measure for further analyses.

### 2.7 Statistical analyses of imaging variables

All the analyses were performed using Resting-State fMRI Data Analysis (REST) (Song et al., 2011) and corrected with the Gaussian random field (GRF) theory (voxel *p* < 0.001 and cluster *p* < 0.05) for multiple comparisons.

First, to identify the atrophy patterns of left and right-sided SD, the GMV images were compared using two-sample *t*-tests between each of SD groups and NC.

Next, to determine the critical regions of semantic processing in left and right-sided SD patients, we correlated the GMV images with the semantic PCA scores controlling age, gender and education in these two groups, respectively. Furthermore, total GMV and MMSE scores were used as nuisance covariates to eliminate their potential bias. In order to explore the influence of floor and ceiling effects on our data, the mean volumes of significant clusters in right and left-sided SD groups were further compared among three groups using one-way analyses of variance.

## 3 Result

### 3.1 Demographic and neuropsychological results

Based on the comparison of GMV between left and right ATLs, 18 and 17 cases were classified as left and right-sided SD respectively. As shown in Table 1, the demographic and neuropsychological data were compared among left, right-sided SD and NC groups. There were no significant differences in age, gender and education among groups. Additionally, no difference was observed in disease duration between two patient groups. Compared with NC, left and right-sided SD patients presented with impairments on the tasks of general cognition, episodic memory, semantic memory, social cognition and executive function. Moreover, left-sided SD patients showed greater impairments on the picture and definition naming, word reading and Stroop tests than those with right-sided SD. Specifically, left-sided SD group had worse performance of the digit span test than other two groups, whilst right-sided SD group showed impairments on the symbol digit modalities test compared with other two groups. No differences were found on the visuospatial perception, arithmetic, grammar processing and repetition tests among three groups.

The PCA was used to identify the measure of semantics for SD patients. The KMO indexes were >0.5 and the factors captured 70% of variance, which means that the power of our PCA was acceptable. The loading of each test is listed in Table 2. The first factor of left or right-sided SD groups had high loadings on semantic tasks but low loadings on other tests, so we extracted the scores of this factor as the semantic measure of patients for further analyses.

**Table 2:** Factor loadings of each semantic test.

	Right SD	Left SD
	Factor 1	Factor 1
Oral picture naming	0.955	0.936
Oral sound naming	0.695	0.860
Picture associative matching	0.491	0.817
Word associative matching	0.676	0.880
Word-picture verification	0.940	0.920
Naming to definition	0.802	0.855

Note: SD = semantic dementia.

### 3.2 The atrophy pattern of SD patients

**Fig. 1:**
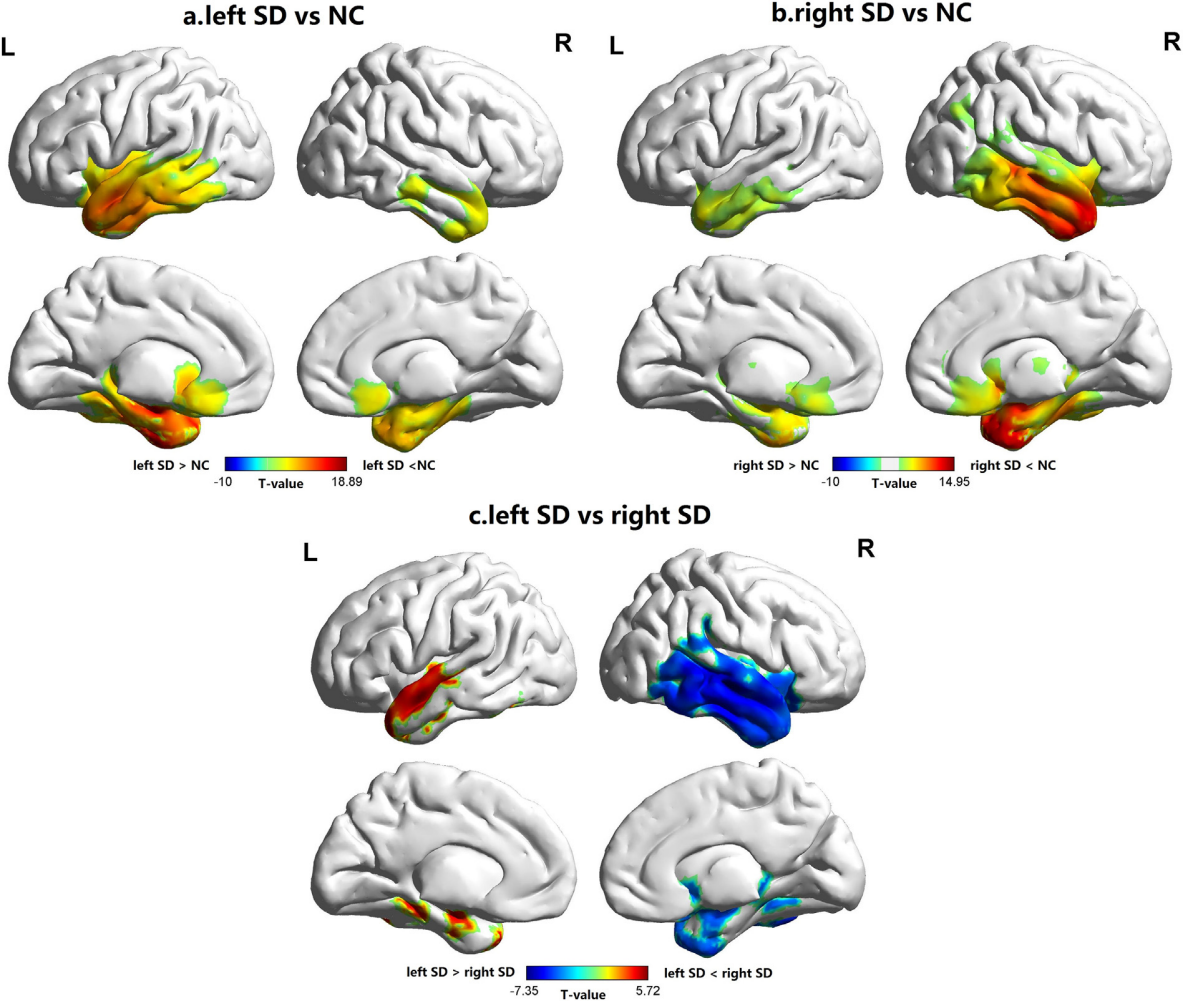
The atrophy pattern of SD patients. a, b and c are the comparison results between left-sided SD vs controls, right-sided SD vs controls, and left SD vs right-sided SD (GRF corrected, voxel *p* < 0.001 and cluster *p* < 0.05). >denotes the group with greater GMV.

Fig. 1 depicted the voxel-based atrophy patterns of left and right-sided SD (GRF corrected, voxel *p* < 0.001 and cluster *p* < 0.05). Compared with NC, both left and right-sided SD groups showed extensive atrophy in bilateral temporal and medial frontal lobes. When two SD groups were compared, left-sided SD group showed circumscribed atrophy in the left temporal lobe on both lateral and medial surfaces, whereas right-sided SD group not only showed greater asymmetric atrophy in the right temporal lobe, but also had widespread atrophy in the right insula, temporoparietal junction and orbital frontal cortex.

### 3.3 The correlations between semantic deficits and brain atrophy

**Fig. 2:**
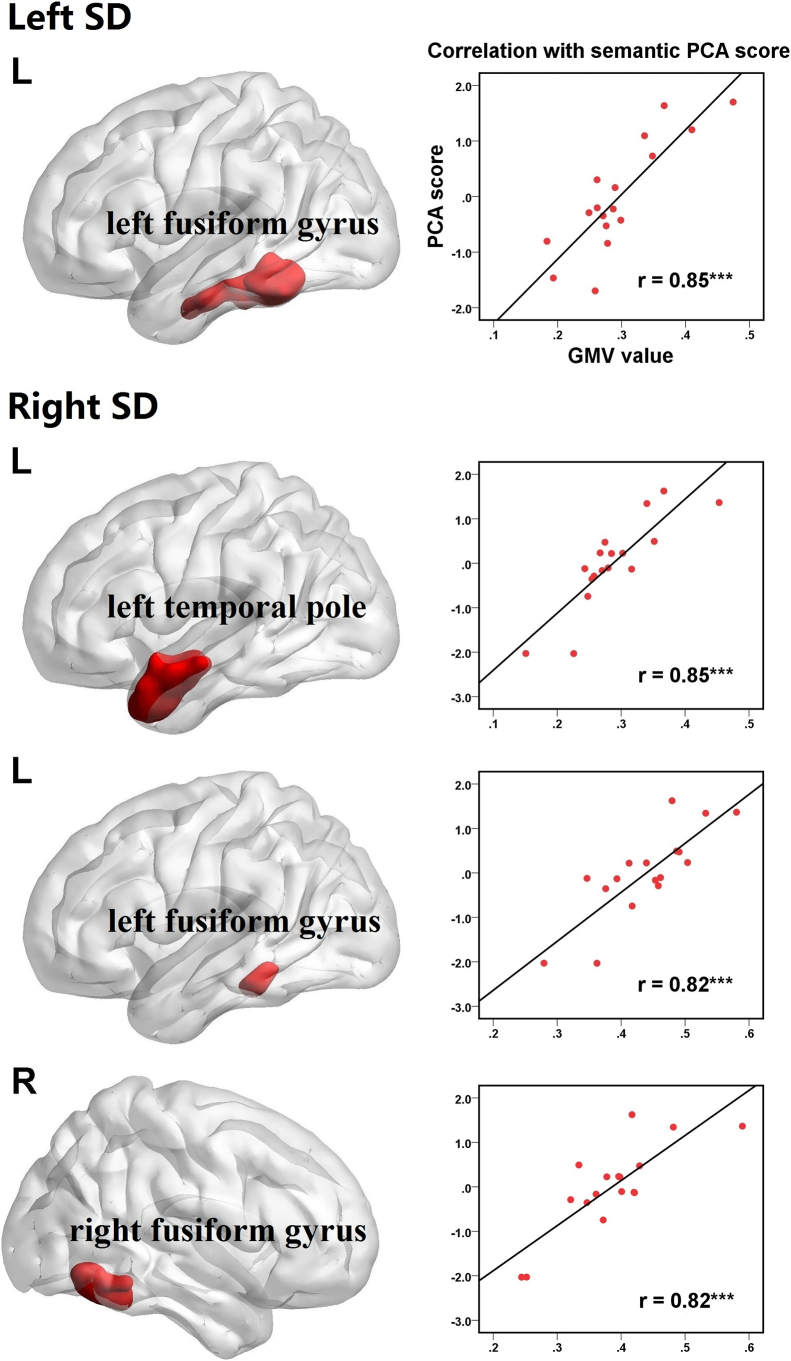
Relationship between GMV value of each SD-semantic related cluster and PCA score (GRF corrected, voxel *p* < 0.001 and cluster *p* < 0.05).

Within the atrophy areas, we further explored the semantic-related regions of left and right-sided SD (see Table 3 and Fig. 2; GRF corrected, voxel *p* < 0.001 and cluster *p* < 0.05). For left-sided SD patients, the correlation analysis identified one cluster in the left fusiform gyrus (cluster size = 2437 voxels; peak coordinates: −34,-46,-16; *r* = 0.85, *p* < 0.001). As to right-sided SD group, three clusters were associated with the semantic deficits, including the left temporal pole (cluster size = 2789 voxels; peak coordinates: −55,-1,-19; *r* = 0.85, *p* < 0.001) and bilateral fusiform gyri (left fusiform gyrus: cluster size = 557 voxels; peak coordinates = −25,-42,-16; *r* = 0.82, *p* < 0.001; right fusiform gyrus: cluster size = 551 voxels; peak coordinates = 46,-61,-9; *r* = 0.82, *p* < 0.001). To exclude potential bias, total GMV and MMSE scores were controlled when correlating mean signals of significant ROIs with semantic deficits. All the effects remained significant (*r* > 0.74, *p* < 0.001). To investigate why the effects of the left temporal pole and right fusiform gyrus were not found in left-sided SD patients, we further compared these ROIs' volumes among SD and NC groups. The results clearly revealed that left-sided SD patients had floor and ceiling effects in these two areas, respectively. They exhibited greater atrophy in the left temporal pole than right-sided SD and NC groups (NC = 3.93 ± 0.43; left-sided SD = 1.78 ± 0.62; right-sided SD = 2.71 ± 0.63; F (2, 51) = 70, *p* < 0.001) whereas showed comparable volume to NC in the right fusiform gyrus (NC = 0.96 ± 0.10; left-sided SD = 0.95 ± 0.17; right-sided SD = 0.72 ± 0.15; F (2, 51) = 17, *p* < 0.001).

**Table 3:** The correlations between semantic deficits and brain atrophy.

Cluster	Brain regions	Cluster size (voxels)	Peak coordinates			*r*	*p*
			x	y	z		
Left SD							
1	Left fusiform gyrus	2437	−34.5	−46.5	−16.5	0.847	<0.001

Right SD							
1	Left temporal pole	2789	−55.5	1.5	−19.5	0.853	<0.001
2	Left fusiform gyrus	557	−25.5	−42	−16.5	0.816	<0.001
3	Right fusiform gyrus	551	46.5	−61.5	−9	0.816	<0.001

Note: SD = semantic dementia.

## 4 Discussion

The current study aimed to compare left and right-sided SD patients systematically and identify the semantic-related regions of right-sided SD. Using a large sample of mild and well-matched SD patients, we found left and right-sided SD patients had comparable deficits in semantic, episodic and general cognitive abilities and common atrophy in bilateral temporal and orbital frontal lobes. Patients with left-sided SD presented with greater impairments of naming, reading, Stroop and digit span tests and more atrophy in the left temporal lobe. In contrast, right-sided SD exhibited more deficits of the symbol digit modalities task and more atrophy in the right frontotemporal lobe. Moreover, we found the left fusiform gyrus correlated with the semantic performance of left-sided SD, whilst the semantic deficits of right-sided SD patients were related to the bilateral fusiform gyri and left temporal pole.

After matching the disease duration, general cognitive and semantic ability, both left and right-sided SD groups showed widespread bilateral temporal and slight orbital frontal atrophy, which reflects SD is a temporal-variant of frontotemporal lobar degeneration (Brambati et al., 2009). When comparing two SD sub-groups, two groups showed greater atrophy in their predominant side respectively, but right-sided SD patients presented with more extensive and severe atrophy than left-sided SD patients, which is in line with prior studies (Chan et al., 2009; Luzzi et al., 2017). This situation might arise from the distinct functions of bilateral ATL (Ralph et al., 2017). Greater damage of the left ATL would lead to more cognitive and language problems and let left-sided SD patients see doctors in earlier time. Regarding the relationship between brain lateralization and cognitive functions, three possible mechanisms can be drawn: (a) Bilateral regions contribute equally to one function; (b) Both sides are linked with one function, but their contributions are quantitatively different; (c) A function completely relies on one side. According to our results, we provide evidence for the anatomical mechanisms of various cognitive functions: (1) The comparable impairments of two SD groups such as general cognition, episodic memory, semantics and social emotion might be based on bilateral frontotemporal lobes equally, which satisfies assumption a. Specifically, these functions might be related with different frontotemporal regions. General cognition might be associated with general atrophy degree. Episodic memory is possibly linked with bilateral medial temporal lobes (Dickerson and Eichenbaum, 2010). Semantics might be represented in bilateral ATL (Ralph et al., 2017). Social emotion might be underpinned by bilateral orbital frontal cortex (Bechara et al., 2000; Kumfor et al., 2013). (2) Some tasks were impaired in both two groups, but showed more deficits in left-sided SD patients, such as reading, naming and Stroop tests. These results imply that left side is engaged more in these functions (i.e. assumption b). Indeed, all these tasks are verbal related, indicating that the left temporal lobe is involved more in verbal processing (Gainotti, 2014; Ralph et al., 2017). (3) If tasks were only impaired in one patient group, it would means these functions are purely driven by one side (i.e. assumption c). Our results suggest some simple language-related tasks such as digit span are related with the left temporal lobe, probably, relying on the posterior temporal lobe with no atrophy in right-sided SD group (Gainotti, 2015; Thierry et al., 2003). On the other hand, the symbol digit modalities task was only impaired in right-sided SD group, suggesting that this primary nonverbal function might be related with the right posterior temporal lobe without damage in left-sided SD group (Gainotti, 2015; Thierry et al., 2003). (4) Other functions, such as visuospatial, calculation, repetition and grammar abilities, were not impaired in both patient groups, indicating that they had no relationship with frontotemporal lobes atrophy (Arsalidou and Taylor, 2011; Mesulam et al., 2014; Saur et al., 2008; Thiebaut De Schotten et al., 2011).

Using separated patient groups, we can avoid ceiling or floor effects and offer the chance to reveal all potential regions related with semantic processing. We found the effect of the left fusiform gyrus in both groups and effects of the right fusiform gyrus and left temporal pole in only right-sided SD group. In fact, a revised semantic theory, unified model, has been proposed during these years (Ralph et al., 2017). It assumes that bilateral ATL are both the hub of semantics, but due to differential connectivity patterns, the sub-regions in the ATL show graded changes of functions. Our findings provide new insights into this theory. First, not only the ATL, but also the fusiform gyrus can be added into this model, because they are both involved in semantic deficits of SD (Mion et al., 2010). Second, the functional diversity appears in two dimensions (i.e. anterior-posterior & left-right directions). After overcoming floor and ceiling effects, only the left temporal pole and bilateral fusiform gyri were found. This result implicates that the role of fusiform gyrus is different from the temporal pole. Studies with NC and SD patients have demonstrated posterior ventral temporal regions are engaged in processing of basic concepts (Bonner and Price, 2013; Hoffman et al., 2015) and tended to work bilaterally. Nevertheless, anterior ventral temporal regions would underpin the processing of specific concepts (Gainotti, 2007; Grabowski et al., 2001; Tranel, 2006) and bilateral parts tend to process different functions. For example, the left ATL is involved in specific object naming (Grabowski et al., 2001; Tranel, 2006), but the right ATL is involved in face recognition (Gainotti, 2007; Rossion et al., 2012). Here, the insignificant results of the right temporal pole might be due to its specific role in face processing, which is not measured in our analyses.

In addition, one interesting finding is that our sample was composed of more right-sided SD patients (50%) than the expectation (25%; Hodges et al., 2010). We speculate that this is because only mild cases (MMSE >18) were recruited in this study. For SD patients, semantic impairments usually do not affect their daily life severely at early stage, so behavioral problems are their important reason to see doctors in our hospital. At early stage, there were few behavioral symptoms in left-sided SD patients. Thus, our sample would be lack of many mild left-sided patients.

Several limitations should be noted. First, we assumed that all patients represented language at the left hemisphere. Indeed, language might be underpinned by right hemisphere or both hemispheres for some subjects. Therefore, further studies should detect the predominant hemisphere of language with fMRI or ERP to exclude potential influence of this point. Second, prior studies have revealed various functions associated with right hemisphere, such as recognition of famous faces and landmarks, social cognition, emotional behavior and non-literal comprehension. These functions might be more vulnerable for right-sided SD than left-sided SD. Therefore, future studies can assess these aspects to reveal more characteristics of right-sided SD.

In conclusion, our findings provide strict comparisons between left and right-sided SD patients and novel evidence for the anatomical mechanism of SD's impaired functions. Our results also offer new insights into the current semantic theory, that the semantic-related regions include both anterior and posterior temporal lobes.

## Funding

This study was funded by grants from the National Key R&D Program of China (grant number: 2016YFC1306305) and the National Natural Science Foundation of China to Qihao Guo (grant number: 81171019).

## Declarations of interest

None.

## Appendix A Supplementary data

Introduce of some semantic tests. Image 1
