# Supplementary material for: The neuropsychological profiles and semantic-critical regions of right semantic dementia
Source: Neuroimage Clin. 2018 May 29;19:767–74. doi: 10.1016/j.nicl.2018.05.035 (PMC6041419; doi:10.1016/j.nicl.2018.05.035)
Supplement: Supplementary file 1 — Introduce of some semantic tests. [file mmc1.docx]

**Introduce of some semantic tests:**

1. Oral picture naming: There were 140 items from 7 categories (animals, tools, common artifacts, fruits, and vegetables, large non-manipulable objects, faces, and actions). Each category had 20 items. Subjects were instructed to speak out the name of each picture.

2. Oral sound naming: It contained 36 items including sounds of animals, tools, common artifacts and others (e.g., the sound of thunder). Subjects heard the target sound through headphones and were required to speak out the name of the objects that produced the sound (e.g., thunder).

3. Picture associative matching: There were 70 items from 7 categories above. Each category had 10 items. Each item included 3 pictures that in the same category. These pictures lined in the manner of equilateral triangle. Subjects were required to select which of the two bottom pictures (e.g., tadpole and lion) was most semantically correlated to the top one (e.g., frog).

4. Word associative matching: This test was identical to the picture association matching test except that the pictures were replaced by corresponding written Chinese names.

5. Word-picture verification: There were 70 trials, with 10 trials from each category of the above oral-picture-naming task. In each trial, the picture of target object (e.g., watermelon) presented in two separate parts, once with target word (e.g., watermelon) and once with semantically corresponded words in same category (e.g., apple). Presenting sequence was random, while it was the same among subjects. Subjects were required to judge whether the object and the word were identical. The trial was scored as correct only if it was correct in these two parts.

6. Naming to definition: It contained 70 items from 7 categories in picture naming test described above. For each item, participants heard a description of the object’s definition (e.g., a kind of conical vegetables which rabbits love to eat), and speak out the name of the object (e.g., carrot).

7. Facial verification: Thirty-six items were included. This test was designed to judge whether two faces from different views were from the same person.

8. Oral repetition: Participants were asked to repeat what they heard. Eight words and 4 sentences were used.

9. Word reading: Participants were asked to read 140 Chinese words.

10. Chinese characters reading: This task was used to evaluate whether the patients suffered from surface dyslexia. We selected 24 Chinese phonetic-semantic compound characters, each containing a phonetic radical and a semantic radical. They included 12 regular or 12 irregular characters, which have or don not have the same sound as their phonetic radicals, respectively. Participants were required to read the characters aloud. Surface dyslexia appeared only if the reading presented regularity effect (i.e., reading worse on irregular than regular characters) and made considerable regularization errors (i.e., pronouncing an irregular character as the sound of its phonetic radical).

11. Cookie theft picture description task: This task was designed to examine whether the patients kept intact grammar processing ability during speech production. This was done by the cookie theft picture description task from the Boston diagnostic aphasia examination. Grammar processing ability of a subject was measured as the percentage of reasonable sentences to all sentences (i.e., the number of reasonable sentences / the number of total sentences that he or she spoke out for the picture).
